# An Environment-Adaptive Multi-Channel Ranging Optimization Algorithm Based on a Multi-Objective Evolutionary Model for Multipath Wireless Sensor Networks

**DOI:** 10.3390/s25185851

**Published:** 2025-09-19

**Authors:** Xuming Fang, Zuqin Ji

**Affiliations:** School of Network Security, Jinling Institute of Technology, Nanjing 211100, China; jizuqin@jit.edu.cn

**Keywords:** high-precision positioning, wireless sensor networks, multi-objective evolution, multi-channel RSSI

## Abstract

Recently, high-precision WSN (wireless sensor network) ranging and positioning algorithms based on RSSI (Received Signal Strength Indicator) in complex indoor environments have become a popular research topic. This is because RSSI is easy to obtain and more suitable for the large-scale deployment of WSNs. However, WSN ranging and positioning algorithms using RSSI are severely affected by the presence of noise and multipath effects in complex indoor environments. To reduce multipath effects, a multi-channel ranging algorithm was developed. This algorithm must obtain accurate initial parameter values or the target–reference distance in advance; otherwise, it will fall into local optima. We propose an environment-adaptive algorithm for multi-channel ranging optimization based on an innovative evolutionary model with multiple objectives and an existing adaptive extended Kalman filter. This novel model includes a newly created objective function of the relationship between weighted multi-channel RSSI and node distance, which allows it to achieve globally optimal results without requiring extensive training to obtain accurate initial parameter values or the target–reference distance beforehand. Extensive simulations and experiments show that our proposed algorithm always has much higher ranging accuracy than the existing algorithm, regardless of whether the multi-channel RSSI is regular or the number of paths matches.

## 1. Introduction

GPS (Global Positioning System) cannot achieve high-precision indoor positioning. Therefore, developing methods to achieve high-precision indoor localization has become a research hotspot. To address this issue, WSN (wireless sensor network)-based indoor positioning algorithms have been proposed [1,2,3]. A WSN is an adaptive network composed of many inexpensive nodes [4,5,6], such as wireless transceivers, microprocessors, and sensors capable of measuring temperature and humidity. Wireless transceivers can measure the RSSI (Received Signal Strength Indicator) between nodes, which is often employed by WSN positioning algorithms to estimate distance [7,8,9].

The main localization algorithms that use RSSI are ranging [10], DFS (Doppler Frequency Shift) [11], Radar [12], and fingerprint [13], among others. Fingerprint positioning establishes a fingerprint map by utilizing different locations’ RSSIs and then matching fingerprints to obtain the target position. In the case of a relatively stable RSSI, fingerprint localization can achieve high accuracy. However, RSSI is easily affected by environmental noise and becomes unstable, resulting in a significant decrease in positioning accuracy. Similarly to fingerprint localization, Radar also needs to establish a fingerprint map to achieve positioning. The difference is that Radar does not entail time-consuming fingerprint matching, but a simple linear-time search. DFS utilizes the Doppler effect to estimate the positions of mobile nodes. However, indoor multipath effects can interfere with localization results. A multi-channel ranging technique based on a multipath signal propagation model is developed to eliminate multipath effects on positioning accuracy using RSSIs.

There are also ranging techniques that do not use RSSIs, instead using TOA (time of arrival), TDOA (time difference of arrival), AOA (angle of arrival), joint TOA and AOA estimation, newton-type forward backward compressed sensing for AOA estimation, and others [14,15,16,17,18]. The TOA-based method employs the signal transmission time to calculate the node distance, while the TDOA approach uses the difference between different nodes’ signal arrival times to compute the distance. In contrast to TOA and TDOA methods, the AOA-based approach needs multiple antennas to measure the angle of wireless signal arrival, which is converted into the distance between nodes. Joint TOA and AOA estimation can always obtain the position of the target node by mapping the pair of TOA and AOA to the coordinates of the target node, even in the presence of many obstacles in the propagation channel. In addition, the newton-type forward backward compressed sensing method is used for estimating AOA parameters to locate target nodes, which can reduce time and space to reconstruct signals and converge faster to the optimal estimate. Due to the extremely fast propagation speed of wireless signals, TOA, TDOA, and AOA all require high-precision clock synchronization, which cannot be applied to wireless sensor nodes with limited energy consumption and cost. Ranging technology with RSSIs does not require high-precision clock synchronization, so it is widely applied in WSN localization.

Although RSSI-based ranging technology does not involve additional hardware costs, it is susceptible to indoor multipath effects. Therefore, the diversity of multi-channel RSSIs is used to improve positioning accuracy in multipath environments [19,20,21]. Multi-channel ranging techniques increase the parameter fitting accuracy of the multipath signal propagation model utilizing multiple channels’ RSSIs. However, since the number of fitted parameters is greater than the objective number, this can lead to an ill-conditioned matrix problem in the fitting process, causing the fitting results to fall into local optima. To estimate the globally optimal node distance, we propose a multi-channel ranging optimization (MCRO) algorithm based on a newly established evolutionary model with multiple objectives. This innovative model evolves the fitting parameters of the multipath signal propagation model towards the global optimum by creating a weighted multi-channel RSSI objective function.

Our contributions mainly include the following aspects.

(1) We establish a multi-objective evolutionary model based on the relationship between weighted multi-channel RSSI and node distance. To our knowledge, this novel model is the only one that utilizes the relationship between weighted multi-channel RSSI and node distance to constrain the evolution parameters so that they do not fall into local optima.
(2) Based on this newly created multi-objective evolutionary model, we propose a multi-channel ranging optimization algorithm for wireless sensor networks in multipath environments. Compared with existing multi-channel ranging algorithms, our proposed algorithm does not require extensive training beforehand to obtain accurate initial parameter values and the target–reference distance.
(3) We employ extensive simulations and experiments to verify the proposed algorithm’s effectiveness and efficiency. The simulations and experiments reveal that, regardless of the accuracy of the initial parameter values, our algorithm always achieves higher accuracy than other ranging algorithms.

The paper is divided into six sections. Related work on multi-channel ranging algorithms with RSSIs is described in Section 2. Section 3 provides the formal expression of the optimization problem for multi-channel ranging, while Section 4 presents the proposed algorithm’s detailed implementation steps, and Section 5 analyzes the simulations and experiments. Finally, a summary is provided in Section 6.

## 2. Related Work

Currently, positioning algorithms for WSNs are mainly divided into two categories: those that require ranging [22,23] and those that do not [24,25]. Ranging-based WSN localization algorithms use the angle and distance to calculate the target location, mainly through triangulation [26], trilateration [27], or multilateration [28]. Triangulation converts the anchor angles into distances, which are then used to compute the target position, while trilateration directly utilizes the distances of three anchor nodes to compute the target location. Multilateration is an extension of trilateration but employs maximum likelihood estimation to calculate the target position when there are more than three anchor nodes.

WSN positioning algorithms that are not based on ranging include CPE (convex position estimation), DV-Hop (distance vector-hop), MDS-MAP (multidimensional scaling-map), centroid, and fingerprint positioning [29,30,31,32,33]. In CPE, convex optimization planning is transformed into a localization problem. After planning is complete, the target position, represented by the rectangular range, is determined. DV-Hop first obtains the hop number between nodes and then uses it to represent distance to estimate the target location. MDS-MAP applies multidimensional scaling to fit the relative target position using the connectivity between nodes. The centroid method determines whether the target node is within the range of three anchor nodes, continues to choose another three anchor nodes that contain the target node, and finally utilizes the centroid of the overlapping parts of all ranges to represent the target location. Fingerprint localization typically uses neural networks and machine learning to establish the relationship between RSSI and location, while neural networks establish path mapping between input and output by training RSSIs measured at different locations. Similarly, machine learning discovers the corresponding classification between RSSI and location through data mining. However, both methods require a significant amount of time and are greatly affected by changes in RSSIs.

The low accuracy of WSN localization algorithms that do not require ranging has led to proposals of ranging-based positioning algorithms, which are mainly based on RSSI, TOA, TDOA, and AOA. Of these, the RSSI-based ranging algorithm is easier to implement, so it is widely applied in resource-limited WSNs. Depending on the channel number required during ranging, ranging algorithms with RSSIs are classified as multi-channel and single-channel. Ranging algorithms based on single-channel RSSIs mainly include Radar, LNSM (log-normal shadowing model), Rips, and Landmarc [34,35,36,37]. LNSM computes the target location by establishing a relationship between RSSI and distance. However, there are two issues with this algorithm: One shortcoming is that training requires significant time to obtain a combination of distance and RSSI samples for fitting the path attenuation exponent. The other issue is that the multipath environment actually varies between the various nodes used for training. The path attenuation exponent obtained using this training method describes the “overall multipath environment”, which differs from the actual situation between different nodes, leading to significant deviation in distance estimation. Rips can obtain accurate distances between nodes through radio interferometry, but it is severely affected by multipath effects. Radar first creates a map based on RSSIs and then uses the map to calculate the target location. Similarly, Landmarc also matches the target node’s RSSI with different locations’ RSSIs to obtain the position. However, they are not suitable for multipath environments where the RSSI is variable.

To eliminate multipath effects, multi-channel ranging algorithms using RSSIs have been developed. The algorithm proposed in [38] utilizes the multi-channel RSSIs’ diversity to eliminate multipath effects. However, this algorithm only averages the RSSIs of different channels and does not model the RSSIs of different signal propagation paths. The multipath-distinguishing-based ranging (MUDR) algorithm proposed in [39] can distinguish between RSSIs of different signal propagation paths. This algorithm estimates the distance by fitting the multipath signal propagation model’s parameters for different channels. However, this algorithm utilizes the least-squares method to fit the model parameters, so if the initial parameters are inaccurate, it will fall into local optima, resulting in large distance estimation errors. Additionally, to decrease the deviation of distance measurement results caused by the antenna gain between different nodes, this algorithm needs to determine the inter-node antenna gain through training in advance. To solve this issue, a multi-channel ranging algorithm using reference nodes is proposed in [40]. This algorithm utilizes the reference and target nodes with the same signal propagation model, eliminating the antenna gain parameter from the node distance estimation process. Although this algorithm eliminates antenna gain parameters by using reference nodes, there is often a significant difference in antenna gain between target and reference nodes, resulting in significant distance estimation errors. Additionally, all target nodes need to use reference nodes to participate in distance estimation, so this algorithm is not suitable for deployment in large-scale WSNs.

## 3. Problem Formalization

The multi-channel ranging optimization problem in multipath environments is formalized in this section. Multi-channel ranging optimization uses measured multi-channel RSSIs to eliminate the impact of multipath signal propagation on distance estimation.

During the measurement of RSSIs of multiple channels, WSN nodes can change the frequency of signal transmission. Presently, there are ready-made products that support this feature.

In a multipath environment, *n* denotes the number of signal propagation paths for a node. Without loss of generality, the LOS (Line-Of-Sight) path is assumed to be *d*_1_, and the NLOS (Non-Line-Of-Sight) path is considered *d_i_*, *i* = 2,…, *n*. $\rho_{i}$ represents the *i*-th path’s reflection coefficient, where $\rho_{1}$ = 1, and $\rho_{i}$ < 1. Assuming that there are *m* channels for transmitting signals between nodes, their corresponding frequencies and wavelengths are *f_j_* and $\lambda_{j}$, *j* = 1,…, *m*, respectively.

In a multipath environment, the signal strength measured by a node is the vector sum of signals from multiple propagation paths [41]. The signal strength of *n* propagation paths synthesized from the *j*-th channel is as follows:

(1) $$P\left(x,\lambda_{j}\right)=\left({\left(\sum_{i=1}^{n}\rho_{i}c\lambda_{j}^{2}d_{i}^{-2}\sin\left(d_{i}\lambda_{j}^{-1}\right)\right)}^{2}\right.{\left.+{\left(\sum_{i=1}^{n}\rho_{i}c\lambda_{j}^{2}d_{i}^{-2}\cos\left(d_{i}\lambda_{j}^{-1}\right)\right)}^{2}\right)}^{1/2}$$

where *x* is the parameter set of the multipath signal propagation model, i.e., $x=\left(c,\rho_{2},\ldots ,\rho_{n},d_{1},\ldots ,d_{n}\right)$; $c=\frac{P_{t}G_{t}G_{r}}{{\left(4\pi \right)}^{2}}$; *G_t_* is the sending node’s antenna gain; *P_t_* is the transmission power; and *G_r_* is the receiving node’s antenna gain.

The *j*-th channel’s RSSI is assumed to be *P_j_*. The optimization goal with multi-channel ranging is to obtain the optimal distance estimation by fitting the parameters of the multipath signal propagation model.

## 4. Proposed Algorithm

### 4.1. Existing AEKF

The AEKF (adaptive extended Kalman filter) is better at filtering the RSSI noise that varies with the environment than the traditional EKF (extended Kalman filter) [42]. The AEKF perceives changes in noise through its NSE (noise statistics estimator).

1. Predict state

(2) $${\hat{x}}_{k\left|k-1\right.}=f\left({\hat{x}}_{k-1}\right)$$

(3) $$F_{k}=\left.\frac{\partial f}{\partial x}\right|\begin{array}{c} {\hat{x}}_{k-1} \end{array}$$

(4) $$P_{k\left|k-1\right.}=F_{k}P_{k-1}F_{k}^{T}+Q_{k}$$

where ${\hat{x}}_{k-1}$ is the state estimation of the dynamic system at time step *k* − 1, ${\hat{x}}_{k\left|k-1\right.}$ is the state prediction of the dynamic system at time step *k* − 1, $F_{k}$ is the Jacobian matrix of function $f\left(•\right)$ calculated based on ${\hat{x}}_{k-1},$ $P_{k-1}$ is the expected covariance matrix of the state estimation, and $P_{k\left|k-1\right.}$ is the state estimation covariance matrix predicted according to $P_{k-1}$.

2. Correct state

(5) $${\hat{z}}_{k}=h\left({\hat{x}}_{k\left|k-1\right.}\right)$$

(6) $$H_{k}=\left.\frac{\partial h}{\partial x}\right|\begin{array}{c} {\hat{x}}_{k\left|k-1\right.} \end{array}$$

(7) $$S_{k}=H_{k}P_{k\left|k-1\right.}H_{k}^{T}+R_{k}$$

(8) $$K_{k}=P_{k\left|k-1\right.}H_{k}^{T}S_{k}^{-1}$$

(9) $${\hat{x}}_{k}={\hat{x}}_{k\left|k-1\right.}+K_{k}\left(z_{k}-{\hat{z}}_{k}\right)$$

(10) $$P_{k}=P_{k\left|k-1\right.}-K_{k}H_{k}P_{k\left|k-1\right.}$$

where ${\hat{z}}_{k}$ is the measurement estimate of the dynamic system at time step *k*, $H_{k}$ is the Jacobian of function $h\left(•\right)$ calculated based on ${\hat{x}}_{k\left|k-1\right.},$ $S_{k}$ is the expected measurement estimation covariance matrix, $K_{k}$ is a feedback matrix for state correction, ${\hat{x}}_{k}$ is the system state estimate corrected based on feedback, and $P_{k}$ is the corrected state estimation covariance.

3. Estimate noise statistics

(11) $$R_{k+1}=\left(1-u_{k}\right)R_{k}+u_{k}\left[\varepsilon_{k}\varepsilon_{k}^{T}-H_{k}P_{k\left|k-1\right.}H_{k}^{T}\right]$$

where $\varepsilon_{k}$ is the estimated and actual measurements’ residual, and $u_{k}$ is a forgetting factor.

(12) $$\varepsilon_{k}=z_{k}-{\hat{z}}_{k}$$

(13) $$u_{k}=\left(1-b\right)/\left(1-b^{k+1}\right)$$

where *b* is an amnestic factor between 0.95 and 0.995, which is often set to 0.96 [43].

Equations (12) and (13) are substituted into Equation (11), and the following equation is derived.

(14) $$\begin{array}{l} R_{k+1}=\left(1-\left(1-b\right)/\left(1-b^{k+1}\right)\right)R_{k}\\ +\left(\left(1-b\right)/\left(1-b^{k+1}\right)\right)\left[\left(z_{k}-{\hat{z}}_{k}\right){\left(z_{k}-{\hat{z}}_{k}\right)}^{T}-H_{k}P_{k\left|k-1\right.}H_{k}^{T}\right] \end{array}$$

### 4.2. Established Evolutionary Model with Multiple Objectives

The execution steps of an MOEA (multi-objective evolutionary algorithm) for obtaining the optimal distance estimation between nodes are demonstrated in this subsection, and then an evolutionary model with multiple objectives is established.

The MOEA simulates the process of species evolution to iteratively evolve the model parameters towards the optimal solution based on the created multiple objective functions [44]. In contrast to least-squares optimization, the MOEA does not require precise initial parameter estimates and is not easily trapped in local optima.

Step 1. The algorithm randomly initializes a population ***P****_t_* consisting of *N* individuals and then employs genetic operations (tournament selection, crossover, and mutation) to generate a descendant population ***O****_t_*.

Step 2. The algorithm creates a mixed population ***I****_t_* of 2*N* individuals by merging ***P****_t_* and ***O****_t_* and obtains non-dominated fronts by sorting ***I****_t_* according to the non-dominated set.

Step 3. Until ***P****_t_*_+1_ is filled, the non-dominated fronts are added to a new population ***P****_t_*_+1_ containing *N* individuals.

Step 4. The evolution of the algorithm ends when the number of iterations reaches the set threshold.

The form of the multi-objective evolutionary model is expressed as

$$minimize\;:\;g\left(x\right)=\left(g_{1}\left(x\right),g_{2}\left(x\right)\right)$$

$$subject\;to:e\left(x\right)=\left(e_{1}\left(x\right),\ldots ,e_{w}\left(x\right)\right)\leq 0$$

where g(*x*) consists of two objective functions, *e* represents the constraint conditions, and *x* is composed of several decision variables.

Objective Function 1 searches for the RSSI estimate that best matches the measured multi-channel RSSI by iterating the model parameters in a multipath environment.

(15) $$g_{1}\left(x\right)=\sum_{j=1}^{m}{\left(P\left(x,\lambda_{j}\right)-P_{j}\right)}^{2}$$

where the *j*-th channel’s RSSI estimation is $P\left(x,\lambda_{j}\right),j=1,\ldots ,m$ in a multipath environment.

(16) $$\begin{array}{l} P\left(x,\lambda_{j}\right)=\\ \left({\left(c\lambda_{j}^{2}d_{1}^{-2}\sin\left(d_{1}\lambda_{j}^{-1}\right)+\sum_{i=2}^{n}\rho_{i}c\lambda_{j}^{2}d_{i}^{-2}\sin\left(d_{i}\lambda_{j}^{-1}\right)\right)}^{2}\right.\\ {\left.+{\left(c\lambda_{j}^{2}d_{1}^{-2}\cos\left(d_{1}\lambda_{j}^{-1}\right)+\sum_{i=2}^{n}\rho_{i}c\lambda_{j}^{2}d_{i}^{-2}\cos\left(d_{i}\lambda_{j}^{-1}\right)\right)}^{2}\right)}^{1/2} \end{array}$$

The multi-channel weighted RSSI and the distance between nodes have the approximate expression [45]

(17) $$\frac{1}{m}\sum_{j=1}^{m}s_{j}≃cv^{2}d_{1}^{-2}$$

where *s_j_* represents the weighted RSSI of the *j*-th channel, and *v* represents the speed of signal propagation.

(18) $$s_{j}=f_{j}^{2}P_{j},j=1,\ldots ,m$$

According to the above expression, Objective Function 2 is established, which can constrain the parameter set evolved by Objective Function 1, accelerating their evolution towards the globally optimal direction.

(19) $$g_{2}\left(x\right)=\left|\frac{1}{m}\sum_{j=1}^{m}s_{j}/cv^{2}d_{1}^{-2}-1\right|$$

The multi-objective evolutionary model consists of Objective Functions 1 and 2, which mutually constrain each other to prevent parameter evolution from falling into local optima and to obtain global optima.

$$minimize\;:\;g\left(x\right)=\left(g_{1}\left(x\right),g_{2}\left(x\right)\right)$$

$$subject\;to:minx_{i}\leq x_{i}\leq maxx_{i},i=1,\ldots ,2n$$

$$where:x=\left(c,\rho_{2},\ldots ,\rho_{n},d_{1},\ldots ,d_{n}\right)$$

### 4.3. Proposed MCRO

Algorithm 1 provides detailed execution steps for MCRO.
**Algorithm 1:** MCRO**Input:** Multi-channel RSSI z
**Output:** Optimal distance estimate $\hat{d}$ 1. Set initial values for MOEA parameters 2. Initialize parameters of AEKF
3. Obtain filtered RSSI $\tilde{z}$ based on AEKF and z
4. Calculate weighted multi-channel RSSI $\bar{z}$ using $\tilde{z}$, frequency *f*
5. MOEA generates *N* decision vectors x 6. t=1 7. *t*_max_ = the maximum generations
8. **while** *t* ≤ *t*_max_ **do**
9. Employ x, $\tilde{z}$, and *f* to Compute *N* g_1_
10. Utilize x, $\bar{z}$ to Calculate *N* corresponding g_2_
11. New *N* x is produced by g_1_ and g_2_ 12. t=t+1 13. **end while**
14. *d*_1_ of first x is chosen as $\hat{d}$ with ascending g_1_ 15. **return** $\hat{d}$

**Figure 1:**
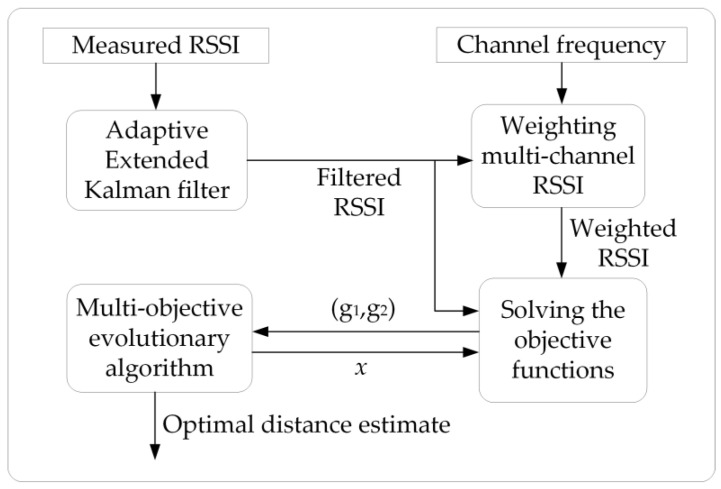
The structure of MCRO.

The proposed algorithm utilizes the evolutionary model with multiple objectives to obtain optimized distance estimates between nodes. Figure 1 reveals the execution process of each functional module in the MCRO algorithm. Firstly, the AEKF module is employed to filter out time-varying RSSI measurement noise. Next, the weighting module utilizes the filtered RSSI to compute the weighted multi-channel RSSI. Finally, the MOEA evolutionary module obtains the optimal distance estimation based on the weighted RSSI.

## 5. Performance Evaluation

In this section, the proposed algorithm’s performance is validated through the results of extensive experiments and simulations, which reveal that MCRO always has higher ranging accuracy than the existing algorithm. This is the case regardless of whether the multipath number is matched or unmatched or whether the multi-channel RSSI is irregular or regular. In order to compare the ranging accuracy of MUDR and MCRO, MUDR also used the same RSSI filtered by AEKF as MCRO in this paper.

### 5.1. Experimental Configuration

**Figure 2:**
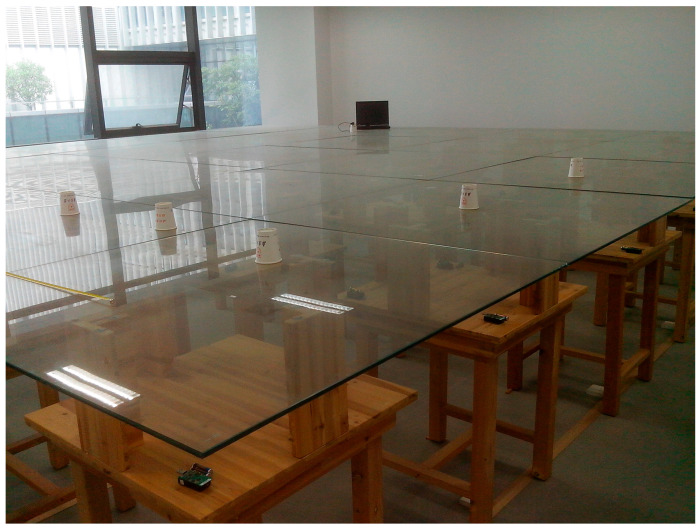
The experimental scenario.

We conducted indoor ranging experiments using TelosB WSN nodes produced by the Crossbow company. The low-cost wireless communication chip CC2420 is installed on the TelosB node, which can provide a continuous available communication bandwidth from 2400 MHz to 2483.5 MHz. We set the channel frequency sequence to *f_j_* = (2405 + 5(*j* − 1)) MHz, *j* = 1, 2,…, 16. The target node sends signals to the beacon node every 0.1 s on 16 channels in sequence, and the beacon node obtains the RSSI value by measuring the signal strength. The target node is randomly placed in an experimental area of 8 by 8 m, as shown in Figure 2. The beacon nodes transmitted multi-channel signals at multiple different distances. Finally, we used the evaluated algorithm to complete ranging.

The values of the parameters are listed in Table 1 for the multi-objective evolutionary model in the experiment. In order to obtain the optimal distance estimation, the number of generations for multi-objective evolution was set to 1000. The parameter values were set based on actual experiments [46]. The parameters and values not listed were set to the most commonly used settings for the multi-objective evolutionary algorithm, and their variations have little impact on the proposed algorithm’s ranging accuracy.

The AEKF’s measurement and state functions are both diag [1,…, 1], and the diagonal matrix diag has the same dimension as the number of channels. Due to the static nature of the nodes, the process noise statistics are set to diag [0,…, 0]. The AEKF can perceive noise, so the measurement noise statistics are set to diag [3,…, 3], and the state estimation covariance is set to diag [1,…, 1]. The AEKF performs 10 time steps each time to filter out measurement noise in the RSSI.

The difference between the node distance estimated by the evaluated algorithm and the actual distance is used to measure the ranging accuracy of the algorithm. Its definition is

(20) $$Ranging\;\mathrm{error}=\left|d-\hat{d}\right|$$

where *d* represents the actual distance between the target node and the beacon node, and $\hat{d}$ represents the distance estimated by the ranging algorithm. The symbol $\left|•\right|$ represents an operator that calculates the absolute value of two numbers.

#### 5.1.1. Influence of Unmatched Multipath Number

**Figure 3:**
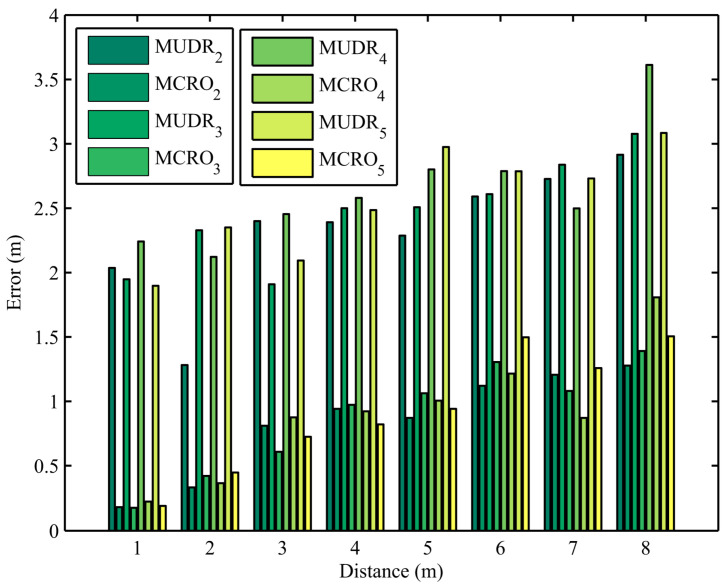
Distance estimation errors of MUDR and MCRO in the experiments using different node distances and path numbers.

Ranging errors obtained from 960 experiments using the MUDR and MCRO algorithms with different multipath numbers and node distances are depicted in Figure 3, where the number of paths and node distances are set to 2 to 5 and 1 m to 8 m, respectively. The subscript of the algorithm name represents the number of paths. Because the path number in the experiment is unknown, the path number set in the algorithm is usually not equal to the actual path number. Since the RSSI vectors of different path numbers in the multipath signal propagation model are equivalent, different path numbers can be used to estimate the same node distance. From the figure, it can be seen that regardless of whether the set number of paths is the same as the actual number of paths, the proposed algorithm’s ranging accuracy is always much higher than that of the existing algorithm, even if ranging errors increase with the distance between nodes.

#### 5.1.2. Influence of Irregular Multi-Channel RSSI

**Figure 4:**
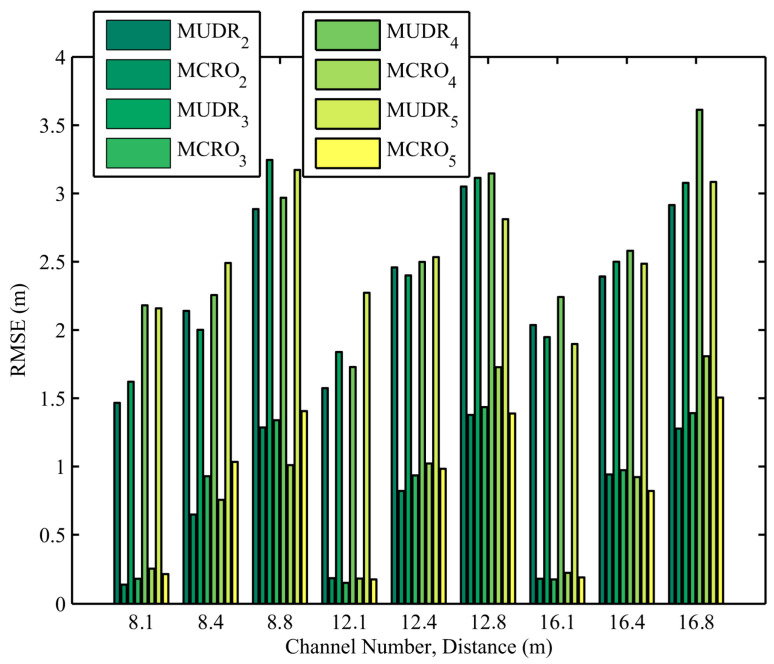
Distance estimation errors of MUDR and MCRO in the experiments using different node distances and channel numbers.

The distance estimation errors of MUDR and MCRO with different channel numbers and node distances in 1920 experiments are depicted in Figure 4. In the actual experiment, the RSSIs measured in multiple channels are irregular compared with the simulated ones. The channel numbers are set to 8, 12, and 16 to observe the impact of the channel number on the distance estimation accuracy. As shown in the figure, the irregular RSSI increases the ranging error of the evaluated algorithm compared to the simulation. However, due to the addition of Objective Function 2 to constrain the relationship between the evolutionary parameters in our proposed algorithm, it always has a much higher ranging accuracy than the other algorithm, regardless of how the channel number changes.

### 5.2. Simulation Configuration

We ran simulations with the algorithm using Matlab to observe the influence of matching path numbers and regular channel RSSIs on ranging accuracy. In the simulation, the distance between the target and beacon nodes is set within 8 m, and the antenna height is set to 0.5 m. The frequencies of 16 channels are set to *f_j_* = (2405 + 5 (*j*−1)) MHz, *j* = 1, 2,…, 16. In addition to the shortest communication path between nodes, multiple reflection paths randomly generated in multipath environments are also added; that is, there is at least one reflection signal path in addition to the direct signal path. In order to obtain optimized results, the number of evolutionary generations was set to 1000. The measurement noise of multi-channel RSSIs is set to follow a normal distribution with a standard deviation of 1. Due to AEKF’s ability to perceive time-varying noise, the initial measurement noise covariance is set to diag [3,…, 3]. The values of other parameters for the MOEA and AEKF in the simulation are the same as in the experiment.

#### 5.2.1. Influence of Matched Multipath Number

**Figure 5:**
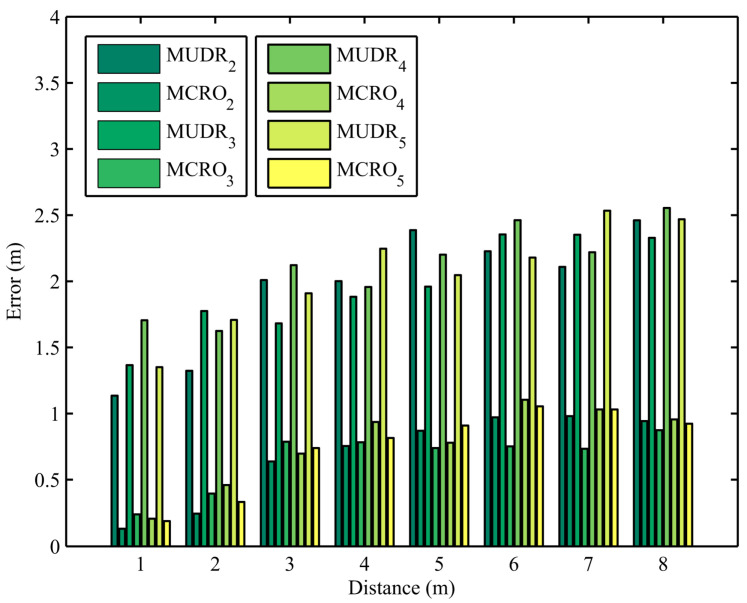
Distance estimation errors of MUDR and MCRO in the simulations using different node distances and path numbers.

The ranging errors of 960 simulations of MUDR and MCRO using different path numbers and node distances are revealed in Figure 5. In the simulations, the path number between nodes is set to two, three, four, and five. Because the path number in the simulation is known, we set the algorithm parameters to be the same as the number of simulated paths to observe the impact of different path numbers on ranging accuracy under matching path numbers. The use of matching path numbers is mainly intended to reduce the impact of path number mismatch on the algorithm evolution results, in order to observe the influence of different path numbers containing different numbers of evolution parameters on the algorithm results. In the figure, it can be observed that, compared with the situation where the algorithm runs with different numbers of mismatched paths in the experiment, due to the matching of path numbers in the simulation, the final evolved parameters are more in line with the model, leading to the evaluated algorithm obtaining more accurate distance estimates. Moreover, due to the added constraints on the model parameters in the proposed algorithm, regardless of how the path number changes, our algorithm always achieves a more optimized node distance estimation.

#### 5.2.2. Influence of Regular Multi-Channel RSSI

**Figure 6:**
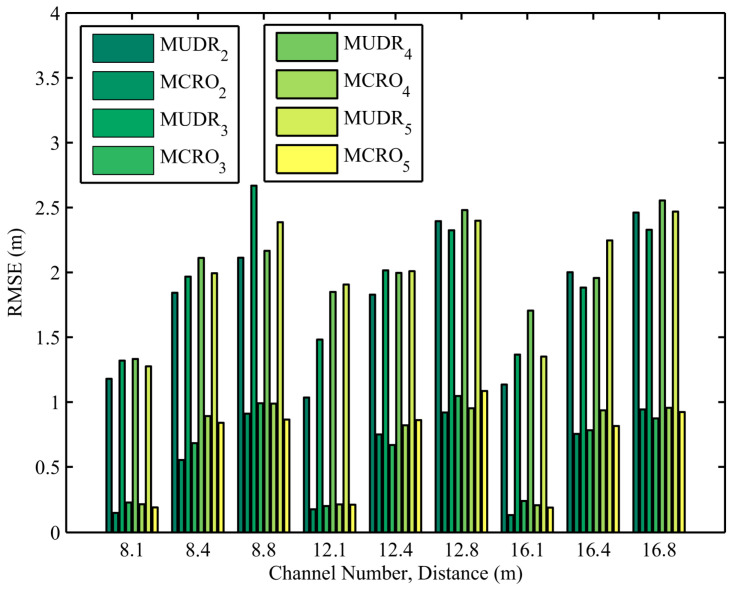
Distance estimation errors of MUDR and MCRO in the simulations using different node distances and channel numbers.

The ranging errors obtained from 1920 simulations of MUDR and MCRO using different channel numbers and node distances are presented in Figure 6. To reduce the influence of the number of paths on the distance estimation results, we set the number of simulated and evolved paths to two, three, four, and five. In the simulation, the multi-channel RSSI is generated based on noise following a normal distribution, which is regular, while in the experiment, the noise of the multi-channel RSSI does not strictly follow a normal distribution, which is irregular. Since the simulated multi-channel RSSI is regular, we observed the effect of channel numbers on ranging accuracy by setting them to 8, 12, and 16. From the figure, we can find that increasing the channel number does not significantly improve ranging accuracy. On the contrary, increasing the number of measured channels actually prolongs the execution time of the algorithm. Therefore, even with a moderate number of channels, we can ensure that the proposed algorithm always has a higher accuracy than the existing algorithm.

### 5.3. Computational Complexity Analysis

The evaluated algorithms were implemented based on Matlab in the simulations and experiments, and their average running time was computed from all the experiments and simulations. Because MUDR does not require evolutionary computation to obtain optimized node distance estimates, its average execution time is around 10 ms. MCRO evolves 1000 generations to obtain optimized node distance estimates, so its run time is slightly longer, at around 400 ms. Although the computation time of MCRO has increased, its ranging accuracy is much higher than that of MUDR. Since MCRO is composed of AEKF and MOEA, and MOEA has a much higher computational complexity than AEKF, the computational complexity of MCRO is equivalent to that of MOEA, that is, MCRO’s computational complexity is *O* (*MN*^2^) [47], where *M* represents the objective number and *N* represents the population size. Thus, we can choose a small population size so that the proposed algorithm can achieve real-time goals.

### 5.4. Ranging-Based Trilateration Positioning

We conducted 90 experiments on trilateration positioning using MUDR and MCRO. Anchor nodes and beacon nodes are randomly deployed in an 8- by 8-m area, and they send messages on 16 channels in sequence. The positioning error of trilateration is defined as

(21) $$Positioning\;\mathrm{error}=\sqrt{\left(x_{1}-{\hat{x}}_{1}\right)+\left(x_{2}-{\hat{x}}_{2}\right)}$$

where $\left(x_{1},x_{2}\right)$ represents the actual coordinates of the target node, and $\left({\hat{x}}_{1},{\hat{x}}_{2}\right)$ represents the coordinates of the target node calculated by trilateration.

**Figure 7:**
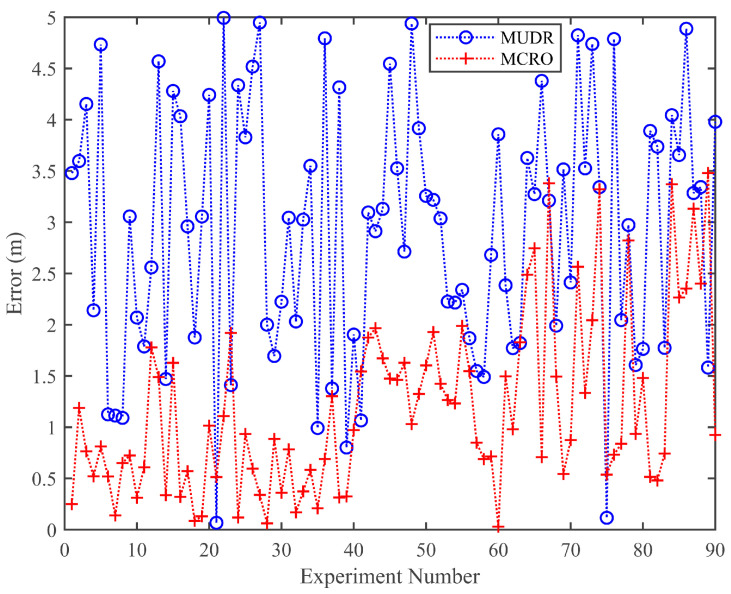
Trilateration positioning error based on MUDR and MCRO in the experiments.

Figure 7 shows the trilateration positioning errors based on MUDR and MCRO in 90 experiments. Due to the unknown number of paths in the actual environment, the number of paths used for MUDR and MCRO is set to five. From the figure, it can be observed that the trilateration positioning error based on MCRO is much smaller than that based on MUDR, with average errors of 1.21 m and 2.92 m, respectively. The main reason for this is that the ranging error of MCRO is much smaller than that of MUDR, which leads to the convergence point of the three sides based on MCRO being closer to the target node.

## 6. Conclusions

In this study, we propose an algorithm for multi-channel ranging optimization based on a novel evolutionary model with multiple objectives. This innovative multi-objective evolutionary model consists of two objective functions: one to evolve the model parameters and the other to constrain the evolved parameters based on the relationship between the weighted multi-channel RSSI and node distance to avoid falling into local optima. MCRO does not need additional training to obtain the path attenuation exponent and node antenna gain when estimating the distance between wireless sensor nodes. It also avoids the problems of reduced accuracy in the ranging model and the need for additional nodes to participate in the ranging process due to the use of reference nodes. Therefore, it achieves the ranging goals of environmental adaptation, high model accuracy, and low deployment cost, and is suitable for the high-precision positioning of nodes in large-scale WSNs in multipath environments. From the simulation and experimental results, it can be concluded that the MCRO algorithm provides more accurate and reliable ranging results compared to the existing multi-channel RSSI-based ranging algorithm, reducing ranging errors. In the experiment and simulation, the average ranging errors of all results decreased by 64%.

## Figures and Tables

**Table 1:** The MOEA’s parameters.

Parameters	Values
max *d*	8 m
min *d*	0 m
max *ρ*	3 × 10^−1^
min *ρ*	0
max *c*	10^−5^
min *c*	0
Generation	10^3^
Population	10

## Data Availability

The data presented in this study are available from the corresponding authors on request.
